# Distribution Patterns of Iron-Oxidizing Zeta- and Beta-Proteobacteria From Different Environmental Settings at the Jan Mayen Vent Fields

**DOI:** 10.3389/fmicb.2018.03008

**Published:** 2018-12-06

**Authors:** Jan Vander Roost, Frida Lise Daae, Ida Helene Steen, Ingunn Hindeness Thorseth, Håkon Dahle

**Affiliations:** ^1^Centre for Geobiology, University of Bergen, Bergen, Norway; ^2^Department of Biology, University of Bergen, Bergen, Norway; ^3^Department of Earth Science, University of Bergen, Bergen, Norway

**Keywords:** iron oxidizers, Zetaproteobacteria, Betaproteobacteria, microbial ecology, hydrothermal systems

## Abstract

Iron oxidizers are widespread in marine environments and play an important role in marine iron cycling. However, little is known about the overall distribution of iron oxidizers within hydrothermal systems, including settings with little hydrothermal activity. Moreover, the extent to which different phylogenetic groups of iron oxidizers exhibit niche specialization toward different environmental settings, remains largely unknown. Obtaining such knowledge is critical to unraveling the impact of the activity of iron oxidizers and how they are adapted. Here, we used 16S rRNA sequencing to characterize the distribution of iron oxidizers in different environmental settings within the Jan Mayen hydrothermal vent fields (JMVFs). Putative iron oxidizers affiliated to Zetaproteobacteria and Betaproteobacteria were detected within iron mounds, bottom seawater, basalt surfaces, and surface layers of sediments. The detected iron oxidizers were compared to sequence types previously observed in patchily distributed iron mats associated with diffuse venting at the JMVFs. Most OTUs of iron oxidizers reoccurred under different environmental settings, suggesting a limited degree of niche specialization. Consequently, most of the detected iron oxidizers seem to be generalists with a large habitat range. Our study highlights the importance of gathering information about the overall distribution of iron oxidizers in hydrothermal systems to fully understand the role of this metabolic group regarding cycling of iron. Furthermore, our results provide further evidence of the presence of iron-oxidizing members of Betaproteobacteria in marine environments.

## Introduction

Zetaproteobacteria were first discovered in iron-rich hydrothermal vents at Loïhi seamount (Moyer et al., 1995; Emerson and Moyer, 2002) and have primarily been detected in hydrothermal systems (Emerson et al., 2007; McAllister et al., 2011; Field et al., 2015; Scott et al., 2015; Makita et al., 2017; Vander Roost et al., 2017). Nevertheless, knowledge of the habitat range of marine Zetaproteobacteria is continuously expanding with the detection of this class in basalts (Henri et al., 2015; Singer et al., 2015), deep-sea shrimp gill chamber surfaces (Jan et al., 2014), pelagic estuaries (Field et al., 2016; Chiu et al., 2017), shallow, coastal peripheries, such as brine-seawater interfaces (Antunes et al., 2011), near-shore bio-corroding steel surfaces (McBeth et al., 2010; Dang et al., 2011; McBeth et al., 2011; McBeth and Emerson, 2016; Mumford et al., 2016; Ramírez et al., 2016), and bay sediments (Laufer et al., 2016, 2017). More recently, members of Zetaproteobacteria have even been detected in a continental context (Crossey et al., 2016).

Current knowledge suggests that all Zetaproteobacteria are specialists in iron oxidation, either using oxygen as an electron acceptor under microaerophilic conditions, or nitrate under anaerobic conditions (Glazer and Rouxel, 2009; Fleming et al., 2013; Kilias et al., 2013; Field et al., 2015; Jesser et al., 2015). Consequently, this class appears to play an important role in the marine iron cycle. Only one Zetaproteobacterial species, *Ghiorsea bivora*, is known to utilize electron acceptors other than Fe(II). Nevertheless members of this species are seemingly restricted to iron-rich environments where they use iron oxidation as their primary energy source (Mori et al., 2017). Globally, Zetaproteobacteria have almost exclusively been observed in Fe(II)-rich environments, with Fe(II) concentrations reaching several hundreds of μM (Scott et al., 2015). A notable exception, however, to this is the recent detection of active pelagic Zetaproteobacteria from Chesapeake Bay, with reported Fe(II) concentrations below 0.2 μM (Field et al., 2016; Chiu et al., 2017). Although low concentrations may not necessarily reflect Fe(II) availability within the Zetaproteobacterial microniches (Chiu et al., 2017), this demonstrates that Zetaproteobacteria may be active in environments with low overall Fe(II) availability.

A significant diversity of Zetaproteobacteria has been documented on a global scale using culture-independent techniques (Kato et al., 2009a,b; Forget et al., 2010; Rubin-Blum et al., 2014; Scott et al., 2015, 2017). However, little is known concerning the connection between diversity and niche specialization, nor how environmental factors may define these niches. Metagenomic studies indicate that oxygen tolerance and nitrogen transformation capabilities may be important factors (Field et al., 2015). Yet the extent to which Fe(II) availability defines microniches has so far not been studied systematically. However, at least some OTUs within Zetaproteobacteria seem to be generalists, growing in a wide range of geographically well-separated environments (McAllister et al., 2011; Scott et al., 2017).

From several sites around the world, 16S rRNA gene sequence types assigned to iron-oxidizing genera within Betaproteobacteria, have been detected together with members of Zetaproteobacteria (Kato et al., 2009b; Li et al., 2012; Meyer et al., 2013; Johannessen et al., 2016; Vander Roost et al., 2017). However, in marine settings, these iron-oxidizing Betaproteobacteria occur in low relative numbers and their presence and possible role in such environments has rarely been investigated. Moreover, given that all cultured Betaproteobacterial iron oxidizers are freshwater organisms, it is unclear as to whether detection of this class within marine settings is merely the result of contamination. However, in a recent study of iron mats from the JMVFs we found no correlation between the relative abundance of Betaproteobacterial iron oxidizers and cell numbers, whereas a positive correlation was found between the relative abundance of Betaproteobacterial iron oxidizers and Zetaproteobacteria. This suggests that the iron-oxidizing Betaproteobacteria are indigenous to the iron mats and not contaminants introduced during sampling or sample processing (Vander Roost et al., 2017).

Although Zetaproteobacteria are commonly detected in hydrothermal systems, their overall distribution across habitats of different hydrothermal activity within the same system has not, as yet, been systematically investigated. Obtaining such knowledge is key to understanding the overall biological cycling of iron in these environments. In two recent studies we documented the presence of Zetaproteobacteria in hydrothermally active areas of the JMVFs (Johannessen et al., 2016; Vander Roost et al., 2017). The aim of the current study was to expand our knowledge of the overall distribution and diversity of Zetaproteobacteria in the JMVFs, including niches with little or no influence of hydrothermal activity. In parallel we further investigated the potential connection between the relative abundance of iron-oxidizing Betaproteobacteria and Zetaproteobacteria and how this may apply to other environmental settings at the JMVFs.

## Materials and Methods

### Sampling Sites

**FIGURE 1:**
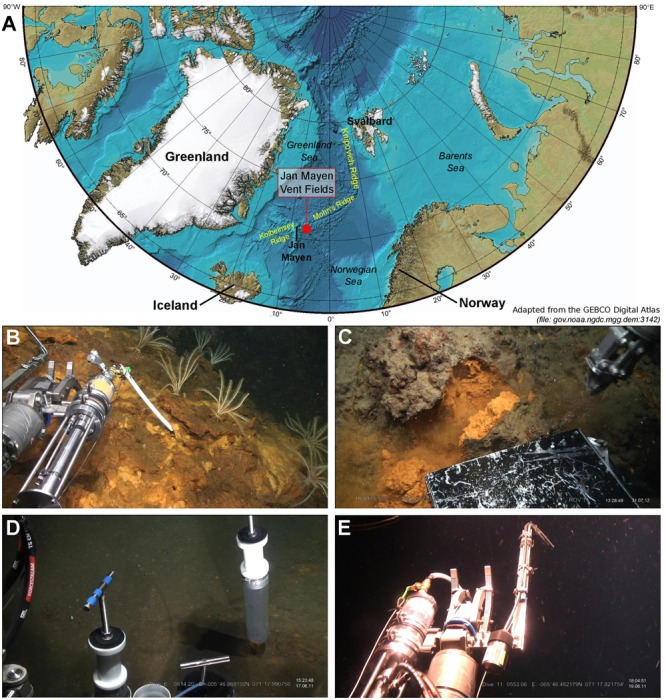
Location of the Jan Mayen vent fields (JMVFs) and images of sampling strategies in this study using a remotely operated vehicle. **(A)** Map showing the location of the JMVFs. **(B)** Sampling an iron mat using a hydraulic suction device. **(C)** Sampling of an iron mound using a shovel-box. Basalt samples were collected with a manipulator arm claw. **(D)** Sampling of sediments using push-cores. **(E)** Sampling of deep-sea water using a hydraulic suction device.

The JMVFs are located at the southern part of the Mohns Ridge in the Norwegian-Greenland Sea (71.2°N, 5.5°W) (Figure 1A), and consist of the Troll Wall Vent Field and the Soria Moria Vent Field. Sampling (Figures 1B–E) was carried out at both vent fields, in which 3–5 cm-thick microbial mats on top of three iron mounds (Mat1-3) (Vander Roost et al., 2017) and samples from the interior of four 30 cm-high iron mounds (IM1-4) were collected. In addition three environments showing no signs of hydrothermal activity were sampled. A sample of basaltic glass with many rusty-colored cracks (Bas) was sampled several hundreds of meters away from any active sites of the Troll Wall Vent Field. In the rift valley of the Troll Wall two sediment cores were obtained: one (Sed1) on the edge of an iron mat and the other (Sed2) from background sediments. The temperature of bottom seawater at the JMVFs is consistently -0.7°C, whereas the temperature at 30 cm below the seafloor (cbsf) in close vicinity of the Sed2 sample was 1.0–1.3°C, indicating very diffuse venting. In the vicinity of the Sed1 sample, the temperature was 56–86°C at 20 cbsf and 1–2°C at ∼1 cbsf, indicating significant hydrothermal activity. The Sed1 sample was 12 cm long and was divided into two subsamples: one at 2 cbsf and one at 20 cbsf. However, no PCR product was obtained from the latter subsample and it was not considered further. The Sed2 sample was 60 cm long and was divided into five subsamples covering a depth-profile going from 0 to 60 cbsf. Lastly, bottom seawater samples were obtained: two replicate samples (SW1-2) from the open water masses 50 m above the seafloor at the Troll Wall Vent Field and one from the seafloor at the Troll Wall Vent Field (SW3). These samples were collected using a hydraulic suction pump connected to a remotely operated vehicle. DNA was extracted and analyzed in duplicate or triplicate per sample, except for the SW3 sample (Supplementary Table 1). In this way, replicates encompass both technical variability and variation between subsamples.

### Chemical Analysis

Once shipboard, pH and alkalinity of the fluids from the hydraulic suction device were measured by a portable pH-meter and an autotitrator (Titrando 888, Metrohm), respectively. Ammonium, nitrate+nitrite, and phosphate were measured photospectrometrically by a Quaatro continuous flow analyzer (Seal). Aliquots for later analyses of major and minor elements by inductively coupled plasma optical emission spectrometry (ICP-OES) (Thermo elemental IRIS) were acidified to 3 vol% of ultrapure HNO_3_ and stored in acid-cleaned HDPE bottles. Both aliquots for ICP-OES and aliquots for anion analysis by Ion Chromatography (Metrohm) were stored at 4°C until on-shore analyses.

### DNA Extraction, 16S rRNA Amplification, and 454 Sequencing

DNA was extracted from around 0.5 g material using the FastDNA^®^Spin Kit for soil (MP Biomedicals, Solon, OH, United States) following the manufacturer’s protocol. Cells were lysed at a speed setting of 6.0 for 40 s with a FastPrep instrument (MP Biomedicals, Santa Ana, CA, United States). 16S rRNA gene amplification was targeted with universal primers for Bacteria and Archaea: Uni787F (5′-ATTAGATACCCNGGTAG-3′) (Roesch et al., 2007) and Uni1391R (5′-ACGGGCGGTGWGTRC-3′) (Turner et al., 1999). The PCR mix consisted of 12.5 μL 2× HotStar Taq master mix (Qiagen), 1 μM of primer, 1 μL DNA template and ddH_2_O up to a total volume of 25 μL. The PCR program initiated with (5′ 95°C), followed by 30 cycles of (45″ 95°C), (45″ 53°C), and (1′ 72°C) and with a final elongation step (7′ 72°C). The size of 16S rRNA gene amplicons was evaluated on a 1.5% agarose gel. Subsequently, PCR products were purified with the MinElute PCR purification kit (Qiagen) following manufacturer’s protocol and eluted with 15 μL ddH_2_O.

Amplicons were tagged in a second PCR, with PCR-mixes containing 10 μL 2× HotStar Taq master mix (Qiagen), 1 μM of MID-barcoded primers, 0.8 ng of template and ddH_2_O up to a total volume of 20 μL. Ultimately, purification of the 16S rRNA-tagged amplicons was achieved by AMPure XP Bead Purification (Agencourt) following the manufacturer’s protocol. DNA quantification was done with a Bioanalyzer (Agilent Biosystems) and equivalent DNA amounts were pooled prior Multiplex GS FLX+/Titanium 454 pyrosequencing (Roche) at the Norwegian High-Throughput Sequencing Centre in Oslo (NHS), Norway and at Microsynth in Balgach, Switzerland.

### Data Collection, Sequence Data Processing, and OTU Analyses

In order to compare 16S rRNA gene amplicons of the current study with previous analyses of iron mats in the Troll Wall Vent Field (Vander Roost et al., 2017), we reanalyzed unfiltered 16S rRNA amplicon sequences (raw sff files available from accession numbers ERS903624, ERS903625, ERS903628, ERS9036233-ERS9036235, ERS903639) together with the sequence data obtained in the current study. Sequence files were processed using the 454 SOP MOTHUR protocol^1^ (Schloss et al., 2011). For sequence filtering, AMPLICONNOISE (Quince et al., 2011) was used as implemented with the “shhh-flows” MOTHUR command. Sequences containing more than six homopolymers were removed. Subsequently, sequences were aligned to the SILVA reference alignment database (silva.nr_v11.align). A screening step required sequences to start before position 2530, and selecting an end position such that 98% of the sequences ended after that position. Chimera-removal was applied by the UCHIME program, implemented in the “chimera.uchime” MOTHUR command. Statistics from the sequence processing are given in Supplementary Table 2. Richness and diversity indices were calculated by the “summary.single” MOTHUR command.

A total of 284,011 high-quality sequences were subjected to taxonomic classification and OTU analysis was carried out on 97% sequence identity level. This resulted in 4957 OTUs after removal of singletons. All sequences assigned to Zetaproteobacteria were extracted from the dataset and compared to predefined “ZetaOtus” using ZetaHunter (McAllister et al., 2011). ZetaHunter was also used for the identification and assignments of ZetaOtus from a previously published dataset of a microbial JMVF mat dominated by Epsilonproteobacteria growing on top of sediments (Lanzén et al., 2012; Urich et al., 2014) (NCBI Sequence Read Archive accession code SRP004929).

Cluster analyses and Non-metric multidimensional scaling (NMDS) analyses were performed using the R package VEGAN (Schloss et al., 2011). For cluster analyses we used the ward.D2 algorithm (Murtagh, 2014) on Bray-Curtis distances. Bubble plots were generated in ggplot2 version 2.2.1 (Wickham, 2009) using the ggplot command.

### Phylogenetic Analyses

For phylogenetic reconstruction of Betaproteobacteria, near full-length 16S rRNA gene sequences (>1200 bp) of *Sideroxydans, Leptothrix*, and *Gallionella* were obtained from the SILVA database^2^. An alignment of these sequences together with OTUs (62 bp) from axial Seamount (Meyer et al., 2013) and OTUs generated in the current study (238 bp) was generated using the online SINA aligner^3^. Based on this alignment a phylogenetic tree was generated in ARB (Ludwig et al., 2004) in two steps: first a backbone tree of the near full-length sequences was generated using the RAxML maximum likelihood algorithm as implemented in ARB. Then, the shorter sequences were added on the tree using the quick-add option in ARB.

For phylogenetic analyses of Zeta- and Betaproteobacterial OTUs used in bubble plot analyses, a maximum likelihood phylogenetic reconstruction was performed in MEGA version 5.2.2 (Tamura et al., 2011), using the Tamura–Nei model (Tamura and Nei, 1993) with default settings.

### Deposition of Sequence Data

Raw sequence data generated through this study have been submitted to the European Nucleotide Archive (ENA) under the accession numbers ERS1737039–ERS1737065.

## Results

### Taxonomy

**FIGURE 2:**
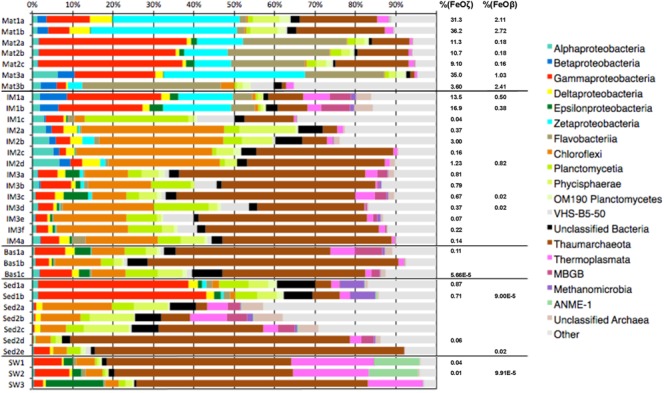
Microbial composition on class level of 34 JMVF samples from five different habitats: Fe mat (Mat), Fe mound (IM), basalts (Bas), sediment (Sed), and deep-sea water (SW). Relative abundances of FeOζ (Zetaproteobacteria) and FeOβ (*Gallionella, Sideroxydans*, and *Leptothrix*) are indicated.

In order to explore the habitat range and distribution of iron oxidizers within the JMVFs, we analyzed samples covering a wide range of habitats, including iron mounds (4 samples), sediments (2 samples), basalts (1 sample), and deep-sea water (2 samples). Results of taxonomic profiling, based on 16S rRNA gene sequencing, were compared to previous analyses of iron mats from the same region (Vander Roost et al., 2017) (Supplementary Tables 1–3 and Figure 2). Overall, the dataset included 284011 reads after filtering (Supplementary Table 2), which were clustered into 4957 OTUs. Putative iron-oxidizing bacteria, i.e., Zetaproteobacteria (FeOζ, 18 OTUs) and members of the Betaproteobacterial genera *Gallionella, Sideroxydans*, and *Leptothrix* (FeOβ, 6 OTUs) were detected in most samples. Apart from the iron mats, relative abundances of these iron oxidizers were low (<3%), with the exception of iron mound 1 (IM1a, b), with relative abundances averaging 10.14 and 0.29% of Zeta- and Betaproteobacteria, respectively (Table 1 and Figure 2).

**Table 1:** Average abundance (%) of the total bacterial community (i.e., bacterial reads), assigned to Beta- or Zetaproteobacteria, at each of the different sites (Mat, iron mat; IM, iron mound; Sed, sediment; Bas, basalts; SW, deep-sea water).

Sample	% (FeOζ)	% (FeOβ)
Mat1	33,72%	2,42%
Mat2	10,36%	0,17%
Mat3	18,53%	1,72%
IM1	10,14%	0,29%
IM2	1,02%	0,21%
IM3	0,49%	0,01%
IM4	0,14%	0,00%
Bas1	0,04%	0,00%
Sed1	0,78%	0,00%
Sed2	0,01%	0,00%
SW	0,02%	0,00%

### Sample Diversity and Distribution of Iron Oxidizers

**FIGURE 3:**
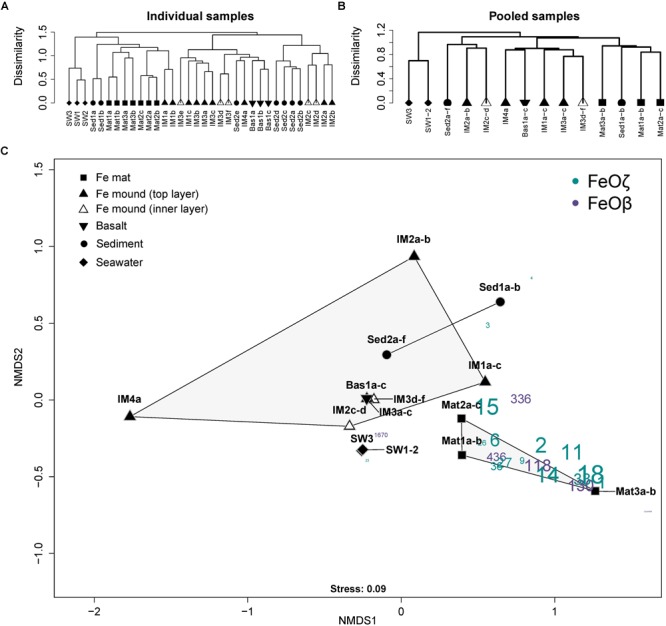
**(A)** Ward.D2 cluster analysis of all microbial communities from the five sampled JMVF habitats. These samples were pooled (highlighted with color) and, consequently, **(B)** subjected to a new cluster analysis. **(C)** NMDS plot situating the pooled samples and the OTU number of the detected iron oxidizers (comprising FeOζ and FeOβ). The FeOζ OTU and FeOβ OTU numbers are scaled to their overall abundance within the entire community.

**FIGURE 4:**
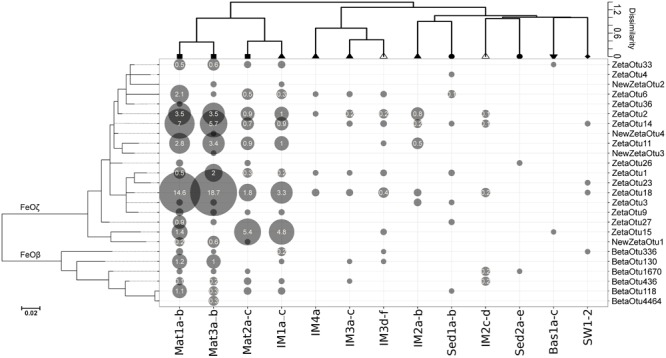
Bubble plot showing the relative abundances of ZetaOtus (FeOζ) and BetaOTUs (FeOβ) across the different habitats (Mat, iron mat; IM, iron mound; Sed, sediment; Bas, basalts; SW, deep-sea water). Samples were pooled as in Figure 3 and clustered by the ward.D2 method using Bray-Curtis distances. Iron oxidizers were phylogenetically clustered by maximum-likelihood. Bubble radii are scaled to their relative abundance within the entire microbial community. Relative fractions >0.1 are indicated in white. Scale bar represents 0.02 substitutions/site.

*Thaumarchaeota* were widely distributed (Figure 2). However, while dominating in the open water masses (SW1-3) and upper parts of iron mounds and sediments, the relative abundance of this group rapidly decreased at increasing sediment depths (Sed2e-Sed2a). Most iron mound samples (IM2-4) and the basaltic glass sample (Bas1) had a class level microbial community composition similar to the open water masses (SW1-3), but with higher relative abundances of *Chloroflexi, Planctomycetia*, and in most cases, Alphaproteobacteria (Figure 2). In one of the iron mounds (IM1) we observed a microbial community more similar to iron mats (Mat1-3) with relative abundances of Zetaproteobacteria reaching up to 17.25%. No characteristic microbial mat structures similar to the Mat1-3 samples were observed by visible inspection of the IM1 sample. However, it cannot be excluded that an elevated flow of hydrothermal fluids occurred at the IM1 sampling site and that an iron mat was under formation. Iron mat samples and deep-sea water samples formed distinct clusters in cluster and NMDS analyses (Figure 3). Most iron mound samples clustered together with basalt samples and sediment samples. Samples from different depths at the same sampling site [0–10 cm below the seafloor (cmbs) for iron mound samples, 0–20 cmbs for sediment samples] generally clustered more closely together than samples from different sites. Hence, there seems to be larger differences in community structure between different mound and sediment sites than there is between different depths in the upper tens of centimeters below the seafloor. Nineteen FeOζ OTUs (classified as “ZetaOtus” by ZetaHunter) and six FeOβ OTUs were identified. Most of these OTUs were found in several locations (Figures 3C, 4). Notable exceptions are FeOζ23, only observed in deep-sea water, and FeOζ4, only observed in sediments. In an attempt to identify FeOζ in hydrothermally more active areas of the JMVFs than the samples considered in Figures 2–4, we investigated a previously published 16S rRNA gene amplicon dataset from a nearby, microbial mat dominated by sulfide-oxidizing Epsilonproteobacteria, growing at the base of an active venting chimney (Lanzén et al., 2012; Urich et al., 2014). Using ZetaHunter we identified four Zetaproteobacterial sequences in this dataset (corresponding to a relative abundance of 0.003%), which all belonged to ZetaOtu18, one of the overall most-dominating FeOζ at the JMVFs (Figure 4). Apart from FeOβ OTU336, all FeOβ observed in iron mats were also identified in iron mounds, basalts or sediment samples (Figure 4).

### Correlation Between FeOζ and FeOβ

Given the notoriously low abundance of FeOβ in marine samples (0–2.72%), it is difficult to exclude the possibility that this group represents contaminants introduced during the extraction of DNA. However, based on an observed significant positive correlation between FeOβ and FeOζ in JMVF iron mats, we have previously argued that this possibility is unlikely (Vander Roost et al., 2017). A similar positive correlation was found when data from the iron mounds, sediments, basalt, and deep-sea water were added (Supplementary Figure 1A). Moreover, a significant positive correlation between FeOβ and FeOζ was also observed when iron mat samples were not included (Supplementary Figure 1B).

### Phylogeny of FeOβ

**FIGURE 5:**
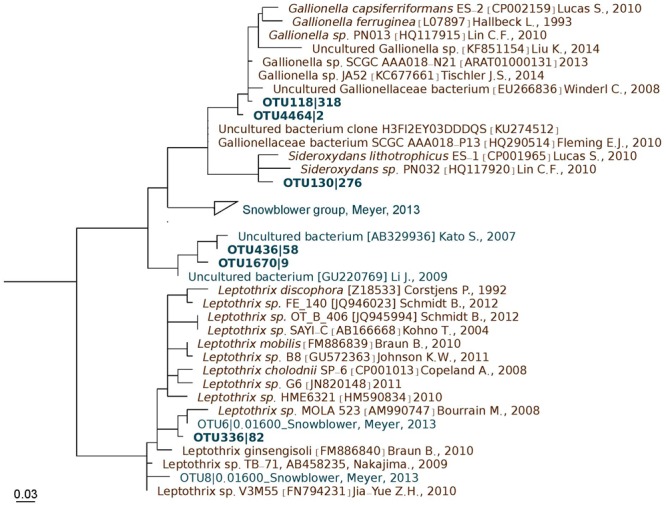
Maximum-likelihood phylogenetic tree of FeOβ from marine (cyan) and freshwater (brown) environments from around the world. Sequences from the JMVFs (this study) are shown in bold. Scale bar represents 0.03 substitutions/site.

Phylogenetic analysis revealed that two of the FeOβ observed in this study (BetaOTUs 436 and 1670) are close-relatives of organisms previously reported in marine environments (Figure 5). Both are close-relatives (>97% sequence similarity) of the marine *Gallionellaceae* clones YS18Uc25 (AB329936) from the Southern Mariana Trough (Kato et al., 2009b) and ELSC-TVG13-B96 (GU220769) from the Lau Basin hydrothermal vent (Li et al., 2012). Three other BetaOTUs (118, 130 and 4464) are more closely related to freshwater strains within *Gallionella* and *Sideroxydans* (Figure 5). A sixth detected FeOβ (BetaOTU336) classified as *Leptothrix* (order *Burkholderiales*) and was identical to a marine FeOβ Illumina-sequenced read (62 bp) from the Axial Seamount, Juan de Fuca Ridge (Meyer et al., 2013).

## Discussion

So far, most analyses of the distribution of Zetaproteobacteria in marine environments have relied on traditional clone libraries of 16S rRNA genes. Given the limitations in sequencing depth associated with this method, it is only suitable for describing the most dominant taxa in a sample. Hence, the presence of some Zetaproteobacteria may have been overlooked in several previous analyses of hydrothermal systems. In this paper, we used high-throughput 16S rRNA gene amplicon sequencing increasing our ability to describe the distribution of iron-oxidizing Zetaproteobacteria (FeOζ) and Betaproteobacteria (FeOβ) in hydrothermal systems. Our results revealed that iron oxidizers comprising both FeOζ and FeOβ classes occur throughout the JMVFs, in iron mounds, sediments, basalts and deep-seawater, albeit at generally low abundance (1.52% on average) (Table 1 and Figure 2). The total abundance of iron oxidizers in these settings arguably outnumbers those in patchily distributed, hydrothermally more active sites where this functional group is abundant. Hence, in order to understand the overall biological impact of iron cycling in hydrothermal systems, it seems to be of importance to take basalts, sediments, mounds, and deep-seawater into consideration.

### Distribution of FeOζ

Almost all FeOζ and FeOβ detected in mounds, sediments, basalts, and seawater were also observed within iron mats. Some notable exceptions are ZetaOtu23 and ZetaOtu4, which were exclusively observed in seawater and sediment, respectively. However, given the limited number of samples analyzed in this study, we cannot conclude that ZetaOtus 4 and 23 are truly unique to these sites. In fact, ZetaOtu4 has previously been reported in various Fe(II)-rich sites around the world, particularly at Loïhi (Scott et al., 2017). Similarly, with the isolation of a member of ZetOtu23, at the Great Salt Bay (GSB2 strain) from mild steel corrosion experiments (McBeth et al., 2011), this might be a generalist representative, showing a cosmopolitan spread and exhibiting opportunistic growth when conditions become favorable. Nearly all detected FeOζ were assigned to predefined ZetaOtus observed outside the JMVFs, indicating that they are not endemic to this region. Given that the detected FeOζ are known to colonize a wide range of environmental settings, this would suggest that most, if not all, of these iron oxidizers should be considered generalists, in the sense that they are able to colonize in a wide range of environmental settings. Interestingly, Scott et al. (2015) did not find any evidence of any specific associations between ZetaOtus and different hydrothermal systems existing under different geological settings (basalt versus ultramafic rock types). This is in line with the detection of cosmopolitan Zetaproteobacteria in three hydrothermal systems on the Mid-Atlantic Ridge (Rainbow, TAG, and Snake Pit). Hence, it appears that a limited number of ZetaOtus reoccur both within and between hydrothermal systems, whereas relative abundance may be determined by environmental factors such as energy availability from Fe(II) oxidation.

With the notable exception of *Ghiorsea bivora* (Mori et al., 2017), members of the Zetaproteobacteria are generally considered obligate Fe(II) oxidizers (Scott et al., 2015). However, based on the global distribution of this OTU, *G. bivora* appears to be absent from H_2_ environments whilst seemingly present under Fe(II) poor conditions. This would suggest that *G. bivora* uses H_2_ only in addition to Fe(II) in natural environments (Mori et al., 2017). Nonetheless, the discovery of *G. bivora* illustrates that Zetaproteobacteria have some metabolic versatility, and we therefore cannot entirely exclude the possibility that at least some of the detected FeOζ may utilize alternative electron donors. Nevertheless, assuming that all FeOζ are obligate iron oxidizers, the observed relative abundance of FeOζ within the JMVFs are consistent with the hypothesis that the relative abundances of different functional groups in hydrothermal systems can largely be explained by the relative densities of energy sources at a given site (Dahle et al., 2015, 2018). We have previously shown that Fe(II) concentrations and Fe(II)/H_2_S ratios are particularly high in the iron mats, presumably due to shallow circulation of hydrothermal fluids in the JMVF rift valley (Johannessen et al., 2016; Vander Roost et al., 2017). Hence, densities of energy available for iron oxidation can be expected to be considerably higher in the iron mats than in surrounding sediments (Vander Roost et al., 2017; Supplementary Table 1). A variable and moderate abundance of FeOζ in more or less active iron mounds is arguably connected to corresponding shifting densities of Fe(II) oxidation energy. These variations stem from the hidden hydrothermal flow paths within iron mounds that shape mound accretion (Johannessen et al., 2016). The lowest fractions of FeOζ were observed in the environments with presumably the lowest densities of potential energy from iron oxidation, i.e., low-temperature deep-sea water and high-temperature sediments with high concentrations of H_2_S.

The detection of Zetaproteobacteria within the water column has previously been reported from a stratified estuary, but to the best of our knowledge, the current study is the first to report the presence of Zetaproteobacteria in deep-sea water. It is plausible that the detected Zetaproteobacteria are not active in this environment, but merely transported from benthic environments to the oxygenated water column through the flow of hydrothermal fluids. Alternatively, and perhaps more in line with the observation of a unique ZetaOtu for this environment (ZetaOtu23), pelagic iron oxidizers may grow in flocs, partly consisting of solid FeS particles which act as a source of Fe(II) and allow the formation of microniches with sharp oxygen gradients. This type of mechanism was recently proposed as an explanation for the presence of pelagic FeOζ in Chesapeake Bay (Chiu et al., 2017).

The first evidence for the capability of Fe(II) in basaltic glass to serve as an electron donor for FeOζ, was demonstrated recently by the growth of ZetaOtu9 (growing up to nearly 40% of the microbial community) on freshly synthesized basalt chips incubated on the abyssal plain of the Atlantic Ocean (Henri et al., 2015; Mori et al., 2017). In more altered, naturally occurring basalts, however, microbial communities are highly diverse, consisting predominantly of *Thaumarchaeota*, a feature that has been related to organic matter contents (Durbin and Teske, 2012). Intriguingly, however, iron-oxidizing microorganisms occur close to or below the detection limit in such environments (Thorseth et al., 2001; Lysnes et al., 2004; Edwards et al., 2011). The current study confirms the presence of Zetaproteobacteria in the basalt samples. Nonetheless, further investigations are needed to reveal how Fe(II) becomes available to the Zetaproteobacterial cell and at what rates they oxidize iron.

### Distribution of FeOβ

A significant correlation was recently identified between the relative abundance of Zetaproteobacteria and Gallionellaceae. This strongly suggests that *Gallionellaceae* represent an intrinsic part of the microbial communities in iron mats of the JMVFs (Vander Roost et al., 2017). In the current study we provide evidence that FeOβ representatives of the genus *Leptothrix* (family *Burkholderiaceae*) may also co-occur with Zetaproteobacteria.

The detection of FeOβ in our samples is somewhat puzzling, given that all isolated members within this group are freshwater organisms. Remarkably, however, three of the detected FeOβ (*Leptothrix* OTU336 as well as *Gallionella* OTU436 and OTU1670) (Figure 4) are close relatives of sequence types previously detected in marine environments. This provides evidence that they belong to distinct marine clades. However, until we manage to obtain pure cultures of marine FeOβ, we can only speculate with regard to their salt tolerance. Moreover, the occurrence of FeOβ at relatively low abundance is also intriguing and will require both more sequencing and cultivation efforts to fully comprehend.

## Conclusion

This study provides evidence that iron-oxidizing Zetaproteobacteria are widespread in both hydrothermally active and inactive sites of the Jan Mayen vent fields. However, we found little evidence for the presence of Zetaproteobacteria endemic to the JMVFs, as most sequences of this taxonomic group were assigned to ZetaOtus previously described in geographically distant areas. The re-occurrence of the same ZetaOtus under highly variable environmental settings across the JMVFs suggests that they are generalists with a broad-habitat range. Our study also provides further evidence for the presence of Fe(II)-oxidizing members of the Betaproteobacterial genera of *Gallionella, Sideroxydans*, and *Leptothrix*, in marine settings.

## Data Availability

Sequencing data in the form of sff.files were obtained from a previous study on iron mats of the Jan Mayen vent fields, deposited at the European Nucleotide Archive (ENA) under sample accession numbers ERS903619-39) (Vander Roost et al., 2017), iron floc samples from the axial seamount, Juan de Fuca Ridge, deposited as part of the VAMPS project (Huse et al., 2014) (https://vamps.mbl.edu/exporter/export_data.php), comprising both JAH_AXV_Bv6 and JAH_AXV_Av6 samples and newly obtained data, submitted under ENA sample accession numbers ERS1737039-65. An overview of the sample names used in this study and corresponding accession numbers are shown in Supplementary Figure 1.

## Author Contributions

JVR collected the sample, performed the lab work, analyzed the data, and wrote the manuscript. FD collected the sample and performed the lab work. IS collected the sample, wrote the manuscript, and supervised the study. IT collected the sample, performed the lab work, supervised the study, and wrote the manuscript. HD collected the sample, performed the lab work, analyzed the data, wrote the manuscript, and supervised the study.

## Conflict of Interest Statement

The authors declare that the research was conducted in the absence of any commercial or financial relationships that could be construed as a potential conflict of interest.

## Footnotes

1 http://www.mothur.org/wiki/454_SOP, accessed April 18, 2017.

2 https://www.arb-silva.de/browser/

3 http://www.arb-silva.de/aligner/
