# Supplementary material for: Distribution Patterns of Iron-Oxidizing Zeta- and Beta-Proteobacteria From Different Environmental Settings at the Jan Mayen Vent Fields
Source: Front Microbiol. 2018 Dec 6;9:3008. doi: 10.3389/fmicb.2018.03008 (PMC6292416; doi:10.3389/fmicb.2018.03008)
Supplement: Supplementary file 1 [file Data_Sheet_1.PDF]

S Table 1: JMVF samples used in this study. * = Accession number, as deposited in the European Nucleotide Archive. § = Temperature measured at 20 cm below the seafloor.

Sample	Accession nr.*	Pooled sample replicates	Sample type	Environment	Sampling method	Coordinates (N'/E')	Depth (mbsl)	First published in
Mat1a	ERS903624	Mat1a-b	Fe mat	Cold-temperature venting at rift valley (T[§]=2-4.5°C)	"Biosyringe" suction sample	71°17.997249/5°46.859220	615.77m	Johannessen *et al.*, 2016
Mat1b	ERS903625							
Mat2a	ERS903633	Mat2a-c	Fe mat	Cold-temperature venting at rift valley (T[§]=2.5°C)	"Biosyringe" suction sample	71°18.002502/5°46.813578	617.67m	Vander Roost *et al.*, 2017
Mat2b	ERS903634							
Mat2c	ERS903635							
Mat3a	ERS903639	Mat3a-b	Fe mat	Cold-temperature venting at rift valley	"Biosyringe" suction sample	71°17.997615/5°46.840857	616.45m	
Mat3b	ERS1737039							
IM1a	ERS1737040	IM1a-c	Fe mound	Mørkdalen	Shovel sample	71°15.123803/5°50.060986	815.45m	
IM1b	ERS1737041							
IM1c	ERS1737042							
IM2a	ERS1737043	IM2a-b	Fe mound: Dark toplayer/crust (0-5mm)		Shovel sample	71°17.985594/5°47.269697	616.89m	
IM2b	ERS1737044							
IM2c	ERS1737045	IM2c-d	Fe mound: Inner, light subsurface layer (5mm-30cm)					
IM2d	ERS1737046							
IM3a	ERS1737047	IM3a-c	Fe mound: Dark toplayer/crust (0-5mm)		Shovel sample		713.62m	
IM3b	ERS1737048							
IM3c	ERS1737049							
IM3d	ERS1737050	IM3d-f	Fe mound: Inner, light subsurface layer (5mm-30cm)					
IM3e	ERS1737051							
IM3f	ERS1737052							
IM4a	ERS1737053	IM4a	Fe mound: Dark toplayer/crust (0-5mm)		Shovel sample	71°17.994864/5°46.824667	614.91m	
Bas1a	ERS1737054	Bas1a-c	Basalt toplayer		Shovel sample	71°17.818957/5°45.689259	455.61m	
Bas1b	ERS1737055							
Bas1c	ERS1737056							
Sed1a	ERS1737057	Sed1a-b	Sediment (20cm depth)	(T[§]=2.5°C)	Pushcore sample	71°17.872160/5°46.364038	567.35m	
Sed1b	ERS1737058							
Sed2a	ERS1737059	Sed2a-e	Sediment adjacent to Fe-mat 1 (60 cm depth)	(T[§]=2-4.5°C)	Pushcore sample	71°17.990756/5°46.868190	567.35m	
Sed2b	ERS1737060		Sediment adjacent to Fe-mat 1 (45 cm depth)					
Sed2c	ERS1737061		Sediment adjacent to Fe-mat 1 (30 cm depth)					
Sed2d	ERS1737062		Sediment adjacent to Fe-mat 1 (15 cm depth)					
Sed2e	ERS1737063		Sediment adjacent to Fe-mat 1 (0 cm depth)					
SW1	ERS1737065	SW1-2	Seawater		Biosyringe' suction sample	71°17.837137/5°46.449153	564.06m	
SW2	ERS1737064					71°17.841765/5°46.447150		
SW3	ERS903628	SW3	Seawater		Biosyringe' suction sample	71°17.821754/5°46.482179	557.25m	Vander Roost *et al.*, 2017
