# Supplementary material for: Distribution Patterns of Iron-Oxidizing Zeta- and Beta-Proteobacteria From Different Environmental Settings at the Jan Mayen Vent Fields
Source: Front Microbiol. 2018 Dec 6;9:3008. doi: 10.3389/fmicb.2018.03008 (PMC6292416; doi:10.3389/fmicb.2018.03008)
Supplement: Supplementary file 2 [file Data_Sheet_2.PDF]

S Table 2: JMVF samples used in this study. * = Accession number, as deposited in the European Nucleotide Archive. ** = Abundance-based coverage estimator as an indicator for sample richness.

Sample	Accession nr.*	Nreads	Coverage	Sobs	ACE**	invsimpson
Mat1a	ERS903624	6582	0.960954	567	928.072577	11.701727
Mat1b	ERS903625	6894	0.960955	613	1330.349032	9.31622
Mat2a	ERS903633	13076	0.960956	800	1857.797141	18.347305
Mat2b	ERS903634	10534	0.960957	674	1523.407833	16.784162
Mat2c	ERS903635	8724	0.960958	636	1453.637764	17.007821
Mat3a	ERS903639	22499	0.960959	906	3618.487367	9.039908
Mat3b	ERS1737039	332	0.96096	79	158.865519	7.771711
IM1a	ERS1737040	10748	0.960961	390	473.507958	34.664642
IM1b	ERS1737041	14543	0.960962	458	518.48594	35.294432
IM1c	ERS1737042	8372	0.960963	893	1291.051698	35.788338
IM2a	ERS1737043	538	0.960964	233	1180.042167	59.715998
IM2b	ERS1737044	601	0.960965	302	2032.747084	82.896552
IM2c	ERS1737045	624	0.960966	203	1025.1875	9.934376
IM2d	ERS1737046	243	0.960967	102	293.569426	9.292984
IM3a	ERS1737047	11883	0.960968	1011	2111.469302	14.474991
IM3b	ERS1737048	1347	0.960969	209	801.318806	7.426869
IM3c	ERS1737049	24312	0.975033	1401	2698.618619	6.160322
IM3d	ERS1737050	17157	0.967477	1253	2544.233844	4.998844
IM3e	ERS1737051	252	0.753968	86	595.107301	9.151042
IM3f	ERS1737052	10016	0.951478	999	2235.002432	5.54831
IM4a	ERS1737053	8428	0.9542	824	1742.264541	6.24828
Bas1a	ERS1737054	936	0.839744	250	767.561936	8.753876
Bas1b	ERS1737055	10340	0.95764	859	2092.781356	3.094101
Bas1c	ERS1737056	17643	0.952502	1781	3696.708914	9.759638
Sed1a	ERS1737057	25608	0.97903	1428	2465.057643	36.813015
Sed1b	ERS1737058	11127	0.967107	912	1680.097165	25.942607
Sed2a	ERS1737059	1067	0.835989	316	876.362411	63.585756
Sed2b	ERS1737060	2303	0.897959	442	1164.323491	24.501812
Sed2c	ERS1737061	1456	0.85783	383	968.229584	67.214925
Sed2d	ERS1737062	1775	0.922254	216	916.333579	4.424133
Sed2e	ERS1737063	4816	0.94539	412	1772.003257	2.892171
SW1	ERS1737065	9510	0.973817	494	1227.259493	6.152088
SW2	ERS1737064	9637	0.97686	457	1099.992419	4.528559
SW3	ERS903628	10088	0.974623	498	1334.280895	5.779677
