# Supplementary material for: Distribution Patterns of Iron-Oxidizing Zeta- and Beta-Proteobacteria From Different Environmental Settings at the Jan Mayen Vent Fields
Source: Front Microbiol. 2018 Dec 6;9:3008. doi: 10.3389/fmicb.2018.03008 (PMC6292416; doi:10.3389/fmicb.2018.03008)
Supplement: Supplementary file 3 [file Data_Sheet_3.PDF]

Supplementary Table 3: Chemical data from the sediment core Sed2. Cmbs = cm below seafloor. ND = not detected.

	Sed2a	Sed2b	Sed2c	Sed2d	Sed2e
cmbs	15	12	9	6	3
pH	6.78	6.78	6.85	6.95	7.38
NH4+ [µM]	10.7	9.89	11.8	6.55	ND
NO3+2 [µM]	ND	6.11	15.0	12.6	27.7
PO43- [µM]	6.52	2.81	2.10	2.88	0.150
Cl- [mM]	561	573		554	570
Br- [mM]	0.78	0.79		0.74	0.78
SO42- [mM]	30.1	30.4		29.0	30.1
Na+ [mM]		399	405	408	404
K+ [mM]		9.62	9.85	10.0	9.72
Mg2+ [mM]	51.0	51.9		52.2	50.9
Ca2+ [mM]	11.4	11.6		10.9	10.3
Si4+ [µM]		696	653	422	168
Sr2+ [µM]		76.2	77.7	78.0	80.0
Li+ [µM]		22.4	23.2	25.0	25.3
Mn2+[µM]		38.9	41.3	28.7	0.287
Fe2+ [µM]		588.4	590.4	273.8	ND
B3+ [mM]		0.382	0.378	0.394	0.421
Ba2+ [µM]		0.150	0.140	0.153	0.157
Co2+ [µM]		0.947¨	1.21	ND	ND
Zn2+ [µM]		2.89	2.67	ND	ND
