# Supplementary material for: Distribution Patterns of Iron-Oxidizing Zeta- and Beta-Proteobacteria From Different Environmental Settings at the Jan Mayen Vent Fields
Source: Front Microbiol. 2018 Dec 6;9:3008. doi: 10.3389/fmicb.2018.03008 (PMC6292416; doi:10.3389/fmicb.2018.03008)
Supplement: Supplementary file 4 [file Data_Sheet_4.PDF]

A

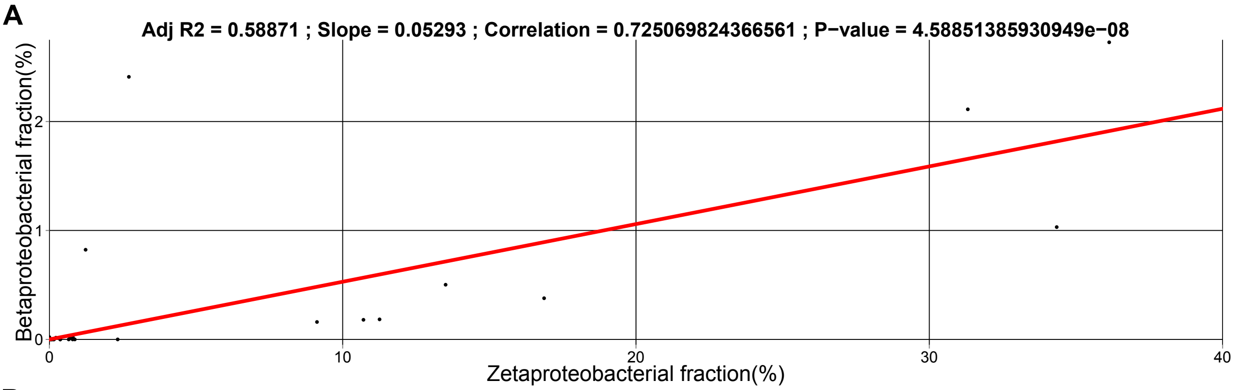

B

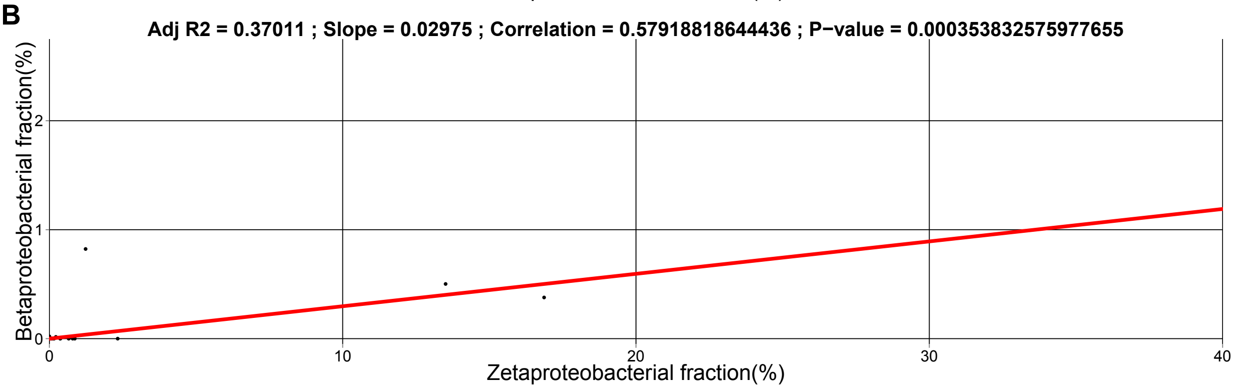

S Figure 1: Correlation between iron-oxidizing iron-oxidzing *Betaproteobacteria* and *Zetaproteobacteria* in all samples (Fe mats, Fe mounds, basalts and sediments (A), and all samples except Fe mats (B).
